# The Operating Room and the Patient

**Published:** 1906-09

**Authors:** 


**The Operating Room and the Patient.** By **Russell S. Fowler**, M.D., Surgeon to the German Hospital, Brooklyn, N. Y. Octavo of 172 pages fully illustrated. Philadelphia and London: W. B. Saunders Company. 1906. (Cloth, $2.00 net).

A book with this title coming at the present time is to be welcomed as an addition of value to the literature dealing with operative preparation. The work is really an expression of Fowler's own ideas regarding operative technic, and is essentially useful also for the purpose for which it was written—namely, that of placing before assistants, internes and nurses, proper and safe methods to be followed in operative technic, preoperative preparation and sterilisation. It is full of valuable instruction to those who have not had the advantages of hospital service.

The book is unique in that it is the first to be published wholly confined to operative technic and preoperative preparation and sterilisation. Not only that, but it outlines preparation of material of all kinds, details necessary instruments for various operations, and describes the preparation of the patient before and his care after operation. There is considerable space devoted to anesthesia which is valuable and instructive, especially that portion which deals with post anesthetic nausea. An interesting feature of the work is the descriptions and illustrations of the different positions of the patient in various operations.

Fowler's style is direct and convincing and his book is happily free from that egotistic straining for effect which mars so many otherwise excellent publications. N. W. W.

